# Method for Correcting Error Due to Self-Heating of Resistance Temperature Detectors Suitable for Metrology in Industry 4.0

**DOI:** 10.3390/s24247991

**Published:** 2024-12-14

**Authors:** Jiyun Li, Hongxing Pei, Orest Kochan, Chunzhi Wang, Roman Kochan, Alla Ivanyshyn

**Affiliations:** 1 College of Modern Information Technology, Henan Polytechnic, Zhengzhou 450046, China; 2 Henan Institute of Advanced Technology, Zhengzhou University, Zhengzhou 450003, China; 3 School of Computer Science, Hubei University of Technology, Wuhan 430068, China; orest.v.kochan@lpnu.ua (O.K.);; 4 Institute of Computer Technologies, Automation and Metrology, Lviv Polytechnic National University, 79000 Lviv, Ukraine

**Keywords:** platinum resistance temperature detector, measurement error, error due to heating by the operating current, error correction

## Abstract

This study contributes to improving the accuracy of temperature measurements with a platinum resistance temperature detector (RTD) by proposing techniques to mitigate the error due to self-heating by the operating current. An assessment of the measurement errors of the platinum RTD was carried out to study ways to improve their accuracy. High accuracy can be achieved by individual calibration using a voltage divider circuit to measure resistance, the substitution method, and the transitional measure. It was shown that each of these approaches offers potential improvements in the accuracy of temperature measurements using RTDs. However, one of the genuine limitations is the error due to heating the RTD by the operating current. To address this, both linear and nonlinear methods for correcting the error due to heating by the operating current were studied. This paper examines how these methods can be applied to mitigate the influence of self-heating on measurement accuracy. Moreover, the residual errors associated with these methods of correction were estimated. The analysis showed that while these methods can reduce the errors significantly, there remain limitations below which it is not possible to mitigate the error.

## 1. Introduction

Industry 4.0 is a new concept that emerged recently. The term “Industry 4.0” itself appeared for the first time in an article published in November 2011 by the German government [1]. This concept implies there is the fourth industrial revolution is now underway, comparable with the first three industrial revolutions [2]. The first industrial revolution was based on the use of steam power, the second was based on the excessive use of electricity, and the third was based on electronics and computation devices [2]. The fourth industrial revolution, referred to as Industry 4.0, involves digital manufacturing, network communication, and computer and automation technologies, as well as security, artificial intelligence, and big data [3,4,5]. However, one of the key factors in all industrial revolutions that moves them forward [6] is measurement accuracy.

Temperature is one of the most frequently measured quantities. The measurement ranges from the vicinity of 0 K to several thousands of degrees. That is why there are many sensors, the most popular among which are thermocouples, thermistors, and temperature resistance detectors [7,8,9,10]. A lot of attention is paid to improving their accuracy, including studies of materials, structures, and metrological procedures [7,8,11].

The temperature resistance detector (RTD) is among the most accurate sensors. Its operation is based on the fact that resistance depends on temperature. Humphry Davy discovered, at the Royal Institution in 1821, that the electrical resistance of metals varies with temperature [12]. Davy’s experiments revealed that when metals are subjected to different temperatures, their electrical resistance changes with respect to temperature. This was a notable result, which broke new ground for future studies of the relationship between temperature and electrical resistance of materials, particularly metals. This, in turn, led to the development of devices like the RTD. Retrospectively, we can consider the discovery of Davy a landmark; however, it was not immediately clear how science and technology could benefit from this effect [12].

The first person who suggested the idea of using the temperature dependence of resistance for a metal to determine temperature was Carl Wilhelm Siemens, a German-born British scientist, engineer, and inventor, who made significant contributions to the field of temperature measurement. Siemens proposed to use a piece of wire and measure its electrical resistance, which could be used as a reliable indicator of temperature. This was a groundbreaking idea at that time. Many important ideas and concepts for designing RTDs were suggested by C. W. Siemens [12].

However, despite all his efforts, early RTDs based on Siemens’ ideas suffered from considerable problems; therefore, they were not accurate enough for accurate temperature measurements. A high price and low accuracy limited the practical application of the RTD [12]. Nevertheless, the simplicity and convenience of estimating the temperature from the resistance of a metal wire inspired further research in this direction.

In 1884, J. J. Thomson was appointed Professor of Physics and Director of the Cavendish Laboratory at the University of Cambridge. His tenure at the Cavendish Laboratory proved to be very successful for physics and culminated with the Nobel prize for his studies of electric current in gases, which led to the discovery of the electron. He also indirectly played an important role in developing the RTD. In autumn of 1885, H. L. Callendar, a new researcher, was hired by Thomson to study the RTD with the goal of improving the design of the existing RTD and making it suitable for accurate temperature measurements [12].

Callendar’s work was highly successful. He achieved considerable gains very quickly, and in the summer of 1886, less than a year after beginning his research, he delivered a report to the Royal Society about his findings. He made significant improvements to the design of the RTD [12]. Platinum was chosen as a material for the sensing element because of its stable and predictable relationship between temperature and electrical resistance. This made it a perfect material for accurate and repeatable temperature measurements.

The success of Callendar’s platinum RTD was important for the field of temperature measurements. It quickly gained popularity in scientific and industrial measurements [12]. Recognizing the importance of this technology, Callendar proposed to establish the standard scale of temperature based on the platinum RTD as an interpolating sensor. His proposal was well received. The International Temperature Scale, which is used today, incorporates the platinum RTD as an interpolation instrument to define temperature values between fixed points on the scale.

In response to these needs, manufacturers began experimenting with new materials and designs for RTDs. One of the most significant advancements was the introduction of thick-film RTDs, which replaced the traditional coil of platinum wire with a thin layer of platinum that was deposited onto a ceramic substrate [9]. This new design made the RTD smaller, more durable, and easier to manufacture in large quantities. Thick-film RTDs also offered improved performance in environments where mechanical shock or vibration might damage a wire-wound sensor.

As the design of RTDs evolved, so too did their applications. Today, platinum RTDs are used in a wide variety of industries, including the automotive, aerospace, food and beverage, pharmaceutical, and chemical industries, where precise temperature control is essential. RTDs are also used in scientific research, particularly in fields like meteorology, climatology, and materials science, where accurate temperature measurement is critical.

The widespread use of platinum RTDs in industrial settings has led to the production of millions of these devices each year. Manufacturers have developed automated processes for producing RTDs, ensuring that they can be made quickly, cost-effectively, and with consistent quality. This has made platinum RTDs more accessible and affordable for a wider range of applications, further increasing their popularity.

## 2. State of the Art

Nowadays, the conventional platinum RTD is considered the most accurate and widely available temperature sensor for measurements from cryogenic temperatures up to 500 °C [9]. Its high accuracy has been confirmed by numerous studies. The potential for high accuracy is underscored by the use of the platinum RTD as a temperature standard in many scientific and industrial settings. One of the key factors in achieving such high accuracy, similar to the standard platinum RTD, is the use of an individual calibration (IC) for each thermometer. This allows the RTD to be finely tuned for specific conditions, ensuring that temperature measurements are as accurate as possible. To further validate this claim, an in-depth analysis of temperature measurement methods using the platinum RTD was conducted, focusing on how IC can improve accuracy. This analysis provides a comprehensive understanding of how the platinum RTD can achieve superior accuracy of temperature measurements in various applications.

Frequently used circuits of unbalanced bridges [13,14,15] for measuring the resistance of the RTD have significant disadvantages: low sensitivity of the bridge circuit, a significant influence on the error of resistances of bridge elements (legs of the bridge, and its power supply circuit), the resistance of lead wires that connect the RTD to the bridge, etc. The only advantage of the bridge circuit is the relatively low requirements for the digital voltmeter or the analog-to-digital converter (ADC), which measures the voltage across the bridge output.

There are circuits that are frequently used to power the platinum RTD from either a current stabilizer or a relatively high voltage stabilizer with a series resistor. The series resistor sets the operating current in the circuit [9,14]. The advantages of such circuits are the practically absent influence of lead wires (in the case of the four-wire connection) and higher sensitivity. However, there are also significant disadvantages: the influence of the power supply circuit on the measurement error and significantly higher requirements for the ADC. Nowadays, however, the latter drawback is insignificant, because 24-bit sigma-delta ADCs [16] are widely available.

**Figure 1:**
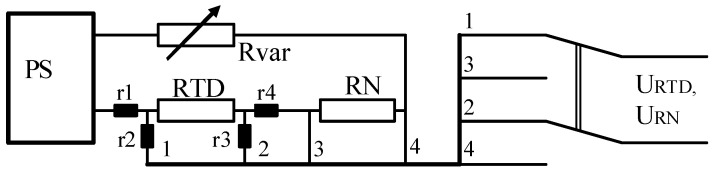
The voltage divider circuit for measuring the RTD resistance.

The voltage divider circuit for measuring the RTD resistance [17,18], presented in Figure 1, has the best metrological properties. The power supply (PS) should ensure short-term stability (during a series of measurements of voltage drops across the resistor *R_N_* and the *RTD*) and a low level of noise. The variable resistor *Rvar* is used to adjust the current in the circuit in such a way as to ensure voltage drops across *RN* and *RTD* close to the upper limit of the measurement range of ADC to minimize quantization error. In this circuit, the RTD’s resistance *R_RTD_* is calculated on the basis of the measurements of the voltage drops *U_RTD_* across the RTD and the voltage drop *U_RN_* across a reference resistor whose resistance is equal to *RN* [17,18]. The lead wires *r*1 … *r*4 in the chosen four-wire circuit do not have any effect on the accuracy of measurements.

(1) $$R_{RTD}=RN(\frac{U_{RTD}}{U_{RN}})$$

From (1) it can be seen that the error of the RTD resistance *δ_RTD_* strongly depends on the accuracy of resistor *RN*. The only factors determining the error of the ratio (*U_RTD_/U_RN_)* are the random error of the ADC and its nonlinearity [14,19]. The resistances of two lead wires (highlighted in black) only influence the measuring current in the circuit, whose instability is accounted for in (1) when measuring the voltage drop across *RN*. The remaining two lead wires (with resistance of the order of tens of Ω) and the ADC’s input resistance (with resistance of the order of tens of MΩ) construct a voltage divider; therefore, their impact is negligible. Thus, the error of the RTD resistance in Figure 1 depends on:

1. The measurement error of the individual resistance $\delta_{RN}^{M}\leq 0.02\%\;(0.05\;{}^{\circ}C)$ of the resistor RN, as well as its temperature $\delta_{RN}^{T}\leq 0.01\%/10\;{}^{\circ}C\;\;(0.025\;{}^{\circ}C)$ and temporal $\delta_{RN}^{\tau }\leq 0.008\%/2000\;h\;\;(0.02\;{}^{\circ}C)$ instabilities [9]. These figures are chosen so that the net error of this resistor has to be at least two times less than the required error of the temperature measurements. Due to relatively short period of measurements (of the order of seconds) its temporal instability can be neglected.
2. The measurement error of the ratio of voltage drops across the RTD and the *RN* is fully determined by ADC nonlinearity and random error [16,19]. For a precision 24-bit sigma-delta ADC, these errors are no greater than 0.0001% (0.00025 °C) and 0.0016% (0.004 °C) for the entire input voltage range. But the resistance of the platinum RTD when measuring the temperature changes within a relatively narrow range, so ADC’s nonlinearity does not exceed 0.002 °C.
3. The normal mode rejection ratio and common mode rejection ratio for 24-bit sigma-delta ADCs are *K_NMR_* ≥ 60 dB and *K_CMRK_* ≥ 120 dB, respectively [16,19,20]. Under laboratory conditions, the influence of interference usually does not exceed 0.002 °C.
4. Error due to parasitic thermal electromotive force in the measuring circuit does not exceed 1 μV (0.002 °C) when using reed relays with a thermal equalizer [21].

The total error of the RTD resistance when measuring it according to the circuit in Figure 1 is equal to 0.06 °C. As can be seen from the analysis above, this error depends mostly on the errors of the reference resistor RN. The magnitude of this error can be mitigated when using the standard resistor of the highest accuracy class to measure the resistance of *RN*. In this case, $\delta_{RN}^{M}\leq 0.013\%\;\;(0.03\;{}^{\circ}C)$, and in the case of active thermostating of *RN*, $\delta_{RN}^{T}\leq 0.001\%/{}^{\circ}C\;\;(0.0025\;{}^{\circ}C)$. So, the total measurement error of the RTD resistance is equal to 0.035 °C.

However, the error of temperature measurement will also include errors of the conversion of the PRT resistance into the temperature, that is, the error of determining its individual conversion function, or, in other words, IC. If one does not use IC, the error of temperature measurement using the platinum RTD with the permissible error of 0.1% will be no greater than 0.3 °C at 0 °C, and 0.5 °C at 100 °C [9]. When using IC versus the third category standard PRT [22], the temperature error will be no greater than 0.1 °C at 0 °C and 0.15 °C at 100 °C.

It should be noted that relative temperature measurements are very common in praxis, in particular, compatible measurements (studying all kinds of dependencies, for instance, the effect of changes in external temperature on temperature gradients in a precision thermostat). In this case, the result of measuring changes in the profile of the temperature field, when the RTD is in series as shown in Figure 1, is practically unaffected by both the resistor errors and errors of IC of the platinum RTD. Then, the measurement error of changes in the profile of temperature field will not exceed 0.006 °C.

But the above figures are correct only without consideration of the error due to heating of the RTD by the operating current. According to [23], this error should not exceed ${∆}_{SN}^{ST}\leq 0.2\;{}^{\circ}C$ if the power dissipated by the platinum RTD is $P_{SN}^{ST}\leq 0.01\;W$. The power dissipated by the platinum RTD depends on many factors, such as the operating current *I_RTD_*, the sensitivity of the ADC (more precisely, its noise and the residual level of interference *U_NZ_*), and RTD sensitivity $S_{RTD}\approx 0.4\%/{}^{\circ}C$. In [24], the optimum RTD current *I_RTD_* was determined. With this current, all contributions of the influencing factors listed above are equal, i.e., their total influence is minimal

(2) $$I_{RTD}=\sqrt[{3}]{\frac{U_{NZ}P_{SN}^{ST}}{{∆}_{SN}^{ST}R_{RTD}^{2}S_{RTD}}}$$

**Figure 2:**
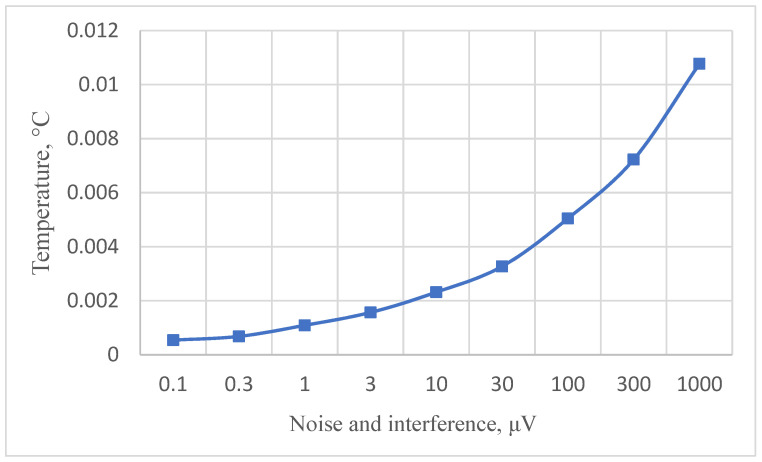
Dependence of the RTD heating temperature on ADC noise.

By substituting into (2) the values of *U_NZ_* from 0.1 to 100 µV, $P_{SN}^{ST}\leq 0.01\;W$, ${∆}_{SN}^{ST}\leq 0.2\;{}^{\circ}C$, *R_RTD_* = 100 Ω, and $S_{RTD}\approx 0.4\%/{}^{\circ}C$, we will obtain the corresponding values of the operating current *I_RTD_* and actual power $P_{RTD}=I_{RTD}^{2}R_{RTD}$ , which is dispersed by the RTD. After substituting the obtained values of *P_RTD_* into proportion

(3) $$\frac{P_{RTD}}{P_{SN}^{ST}}=\frac{{∆}_{RTD}}{{∆}_{SN}^{ST}}$$

we obtain the dependence of the error of the optimal heating of the RTD, with respect to this heating by the operating current, on the random error of the ADC. This dependence is shown in Figure 2.

Figure 2 shows that with the rise in the noise level and residual interference, error due to heating the RTD with the optimal operating current increases considerably. Therefore, during high-precision measurements in harsh conditions, it is reasonable to correct this error. But when conducting high-precision relative measurements even under laboratory conditions (the random ADC error level is up to 10 μV), taking into account the heating of the RTD by the operating current, even when setting its optimal value, becomes necessary.

Methods were developed for correcting self-heating in the platinum RTD [25,26], but they were developed for the highest accuracy measurements using a standard platinum RTD. They do not work for industry because a long period is required to obtain corrected temperature measurements. The method in [25] has the measurement uncertainty of the order of 50 μK. The paper [26] proposes a few possible ways to correct the effect of self-heating of the standard platinum RTD by its operating current. Uncertainties vary from 7 μK to 43 mK. A better performance of the latter is achieved by the method of least squares, which mitigates random errors. Therefore, it is reasonable to use it to estimate zero current resistance.

One of the widely used methods to reduce error due to heating of the RTD by the operating current is to increase this current during the measurement process (i.e., powering the RTD with a pulsed current). However, at the same time, thermal transience occurs, which excites thermal waves in the measured object. For low-inertia measuring objects, this can lead to the appearance of an error of the method, because of distortion of the temperature field, the appearance of transient processes and heat waves, and problems with a temperature control system. Therefore, it is reasonable to develop methods for correcting the error due to heating of the RTD by its operating current.

The aim of this work was to develop a method for correcting the error due to heating the RTD by its operating current. The correction is based on the method of least squares.

## 3. A Simple Method for Correcting Error Due to Heating of the RTD by Its Operating Current

To achieve the aim of the study, preliminary studies of the individual properties of the error at a particular measurement object are needed. In general, heating *T* of a certain body can be described by Newton’s law of cooling, which is a differential equation [27]. Its solution is the exponential dependence of temperature on time. However, in this particular case we are not interested in the function of change in temperature over time. We are interested in the value of temperature rise caused by the power dissipated by the RTD in the steady state, i.e., after the end of the thermal transient. Therefore, the error due to the heating of the RTD by its operating current Δ*_SN_* can be described using the equation that connects it with the cause of its appearance—power *P_RTD_*, which is dissipated by the RTD [8].

(4) $${∆}_{SN}=F(P_{RTD})$$

In general, Equation (4) is nonlinear because the heat transfer of the RTD depends not only on the heat transfer conditions but also on temperatures of the RTD and environment. However, as was shown earlier, error due to heating of the RTD does not exceed hundredths of a degree, so it is possible to consider (4) as a linear function within a narrow range. In this case, (4) it can be rewritten as:

(5) $${∆}_{SN}=KP_{RTD}=KI_{RTD}^{2}R_{RTD}$$

where *K*—the coefficient of heat transfer from the RTD to environment.

Then the actual temperature of the measurement object *T_MO_* can be defined as:

(6) $$T_{MO}=T_{RTD}-KI_{RTD}^{2}R_{RTD}$$

where *T_RTD_* is the temperature measured by the RTD (under the influence of its heating by the operating current).

Expression (6) is a quadratic equation with both an intercept and a linear term. It contains two unknown variables, namely, *K* and *T_MO_*; therefore, to find them, it is necessary and sufficient to measure *T_MO_* using two values of the RTD’s operating current, namely, *I_RTD_*_1_ and *I_RTD_*_2_. Due to the error of heating the RTD by its operating current, we obtain two distinct values of the measured temperature—*T_MO_*_1_ and *T_MO_*_2_. According to the measurement results, we construct a system of two equations of the form (6). Its solution with respect to the coefficient of heat transfer *K* is as follows:

(7) $$K=\frac{T_{RTD1}-T_{RTD2}}{\left(I_{RTD1}^{2}-I_{RTD2}^{2}\right)R_{RTD}}$$

After that, the actual temperature of the measured object *T_MO_* can be determined by substituting *K* into (6).

Let us estimate the error of measuring the object’s temperature Δ*_MO_* using the proposed method of correcting the RTD’s error due to heating by its operating current. The sources of error will be the error due to the nonlinearity of the conversion function (CF) of the ADC Δ*_NL_* and the error due to ADC noise Δ*_NZ_*.

It is obvious that the smaller the differences in the numerator and denominator of (7), the bigger the error of *K*. Therefore, the ratio of currents *I_RTD_*_1_ and *I_RTD_*_1_ should be as large as possible.

When using an ADC with discontinuous CF, if the discontinuities of an ADC do not exceed 0.0015% (equivalent to AD7714), this corresponds to a temperature error of Δ*T* = *δ_NL_/SRTD* ≈ 0.004 °C. The difference in the numerator of (7) for currents *I_RTD_*_1_ = 2 mA and *I_RTD_*_2_ = 0.5 mA will be *T_RTD_*_1_ − *T_RTD_*_2_ ≈ 0.0075 °C. Due to the effect of jump discontinuities on nonlinearity, the difference in the numerator of (7) can be changed maximally from *T_RTD_*_1_ − *T_RTD_*_2_ − Δ*T* ≈ 0.0035 °C to *T_RTD_*_1_ − *T_RTD_*_2_ + Δ*T* ≈ 0.0115 °C, that is, in 0.0115 °C/0.0035 °C ≈ 3.3 times. It is obvious that, in this particular case, the correction is not reasonable. It will inflate the measurement error rather than mitigate it.

When measuring temperature using the sigma-delta ADC, for instance, AD7714 (which has a continuous CF), which works in the circuit depicted in Figure 1, the error Δ*T*, according to [19], can be estimated from the proportion:

(8) $$\frac{2{∆}_{NL}}{{∆}_{NL}^{RTD}}\approx \frac{T_{R}}{T_{RTD1}-T_{RTD2}}$$

where Δ*_NL_* is integral nonlinearity of the ADC; *U_R_* full range of ADC measurement; *ΔU_RTD_* is voltage drop across the RTD when determining *K*; and ${∆}_{NL}^{RTD}$ is ADC’s nonlinearity when determining *K*.

After substituting the corresponding values into (8), for the measurement range, for example, 100 °C, we obtain ${∆}_{NL}^{RTD}\leq 0.25\cdot 10^{-4}\%$, that is, the error of the numerator in (7) is $\frac{{∆T=∆}_{NL}^{RTD}}{S_{RTD}}\approx 6\cdot 10^{-5}\;{}^{\circ}C$, which can be neglected.

Similarly, we estimate the influence of ADC nonlinearity on the denominator of (7). Its error does not exceed 4·10^−4^ °C.

A bigger error of the correction of heating the RTD by its operating current is caused by the ADC’s noise, while the numerator in (7) has the main influence on the error of coefficient *K*. According to [19,20], the noise of AD7714 within the conversion range 0–320 mV does not exceed *U_NZ_* ≤ *1* μV. The analysis similar to the one given above showed that the numerator of (7) will change by about 3% due to such noise. For correction of the error due to heating of the RTD, which does not exceed 0.008 °C, it is acceptable. The residual error after correction of heating the RTD by the operating current can be estimated at 0.00032 °C, that is, this error is reduced by more than two times.

The best results are obtained when using auxiliary measurements of the voltage drop across the RTD for more than two operating currents to correct the error due to heating the PRT using the method of least squares [28,29,30]. To do this, firstly, a system of *m* linear equations of the *n*-th degree with *n* + 1 unknown coefficients *A*_0_ … *A_n_* is constructed. The system also contains the values of powers *X*_1_ … *X_m_*, which are dissipated by the RTD and the values of temperatures measured under these powers *Y*_1_ … *Y_m_*. The method of least squares works when *m* > *n* + 1 [28,29,30], that is, the number of equations in the system is greater than the number of unknown coefficients *A*_0_ … *A_n_*.

The conventional implementation of the method of least squares is too complicated for 8-bit microcontrollers. Therefore, based on [29], a new algorithm for calculating the coefficients *A*_0_, *A*_1_, *A*_2_ of the quadratic polynomial for correcting the error due to heating the RTD based on the results of measurements of temperatures for the currents *I_RTD_*_1_, *I_RTD_*_2_, *I_RTD_*_3_, *I_RTD_*_4_ was developed. For this, according to the values of powers, $P_{1}=I_{RTD1}^{2}R_{RTD1}\ldots P_{1}=I_{RTD4}^{2}R_{RTD4}$ which are dissipated by the RTD under the action of these currents, and the measured temperatures corresponding to these powers *T*_0_, *T*_1_, *T*_2_, *T*_3_ we can calculate intermediate variables:

$${SP}^{2}=P_{1}+P_{2}+P_{3}+P_{4},$$

$${SP}^{2}=P_{1}^{2}+P_{2}^{2}+P_{3}^{2}+P_{4}^{2},$$

$${SP}^{3}=P_{1}^{3}+P_{2}^{3}+P_{3}^{3}+P_{4}^{3},$$

(9) $${SP}^{4}=P_{1}^{4}+P_{2}^{4}+P_{3}^{4}+P_{4}^{4},$$

$$SPT=P_{1}T_{1}+P_{2}T_{2}+P_{3}T_{3}+P_{4}T_{4},$$

$$SP^{2}T=P_{1}^{2}T_{1}+P_{2}^{2}T_{2}+P_{3}^{2}T_{3}+P_{4}^{2}T_{4},$$

$$ST=T_{1}+T_{2}+T_{3}+T_{4}$$

Next, we calculate the intermediate variables to determine the coefficient next to the power squared:

(10) $$\begin{array}{c} A_{2-C1}=ST\cdot SP\cdot SP\cdot SP^{3}+ST\cdot SP\cdot SP\cdot SP^{2}+4SP^{2}T\cdot SP\cdot SP^{2}\\ A_{2-C2}=4SPT\cdot SP\cdot SP^{3}+ST\cdot SP^{2}\cdot SP^{2}\cdot SP+SP^{2}T\cdot SP\cdot SP\cdot SP\\ A_{2-Z1}=SP\cdot SP\cdot SP^{2}\cdot SP^{3}+SP^{3}\cdot SP\cdot SP\cdot SP-SP^{2}\cdot SP^{2}\cdot SP\cdot SP\\ A_{2-Z2}=SP^{2}\cdot SP^{3}\cdot SP\cdot SP-4SP^{3}\cdot SP^{3}\cdot SP^{3}\cdot SP+4SP^{4}\cdot SP^{2}\cdot SP \end{array}$$

This allows calculating the coefficient at the square of the power:

(11) $$A_{2}=\frac{A_{2-C1}-A_{2-C2}}{A_{2-Z1}-A_{2-C2}}$$

Then we calculate the intermediate variables to determine the intercept:

(12) $$\begin{array}{c} A_{0-C1}=ST\cdot SP^{2}\cdot SP^{2}-ST\cdot SP\cdot SP^{3}-SPT\cdot SP\cdot SP^{2}+SP^{2}T\cdot SP\cdot SP\\ A_{0-C2}=A_{2}(2SP^{3}\cdot SP^{2}\cdot SP-SP^{4}\cdot SP\cdot SP-SP^{2}\cdot SP^{2}\cdot SP^{2}) \end{array}$$

and the intercept itself can be calculated as follows:

(13) $$A_{0}=\frac{A_{0-C1}+A_{0-C2}}{A_{0-Z}}$$

Next, we calculate the intermediate variables to find the coefficient next to the linear term:

(14) $$\begin{array}{c} A_{1-C1}=SPT\cdot SP^{2}-SP^{2}-SP^{2}T\cdot SP\\ A_{1-C2}=A_{2}(SP^{3}\cdot SP^{2}-SP^{4}\cdot SP)\;\\ A_{1-Z}=SP^{2}\cdot SP^{2}-SP\cdot SP^{3} \end{array}$$

and the coefficient of the linear term:

(15) $$A_{0}=\frac{A_{1-C1}-A_{1-C2}}{A_{1-Z}}$$

It is noted that the basis of the least squares method is the calculation of differences, so the calculation of dependencies (9)–(15) should be performed with the maximum accuracy that can be implemented by the microcontroller software.

**Figure 3:**
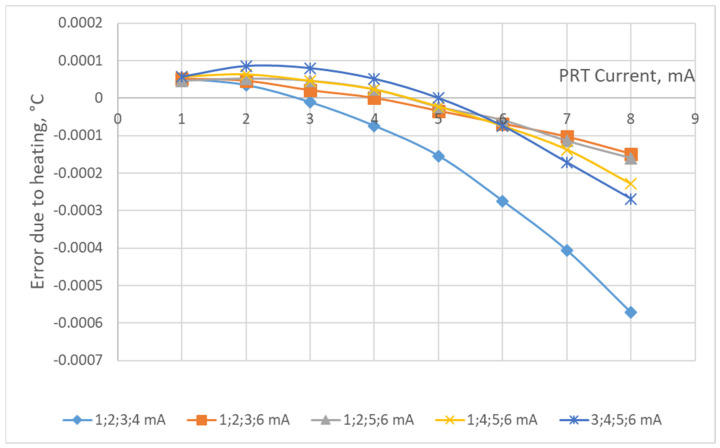
The maximum residual error for four measurements and the maximum ADC noise of 0.0002 °C.

**Figure 4:**
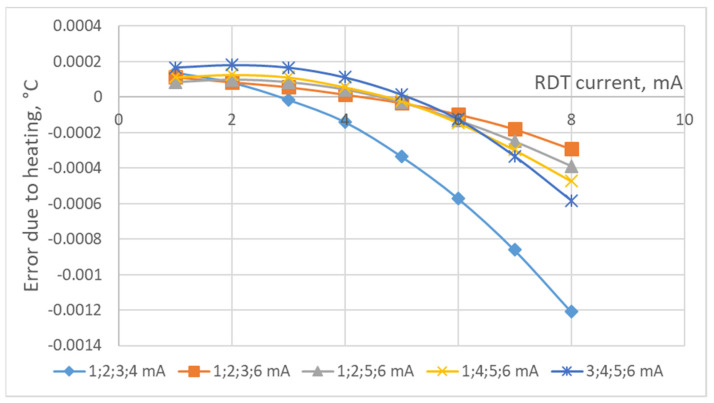
The maximum residual error for four measurements and the maximum ADC noise of 0.0004 °C.

**Figure 5:**
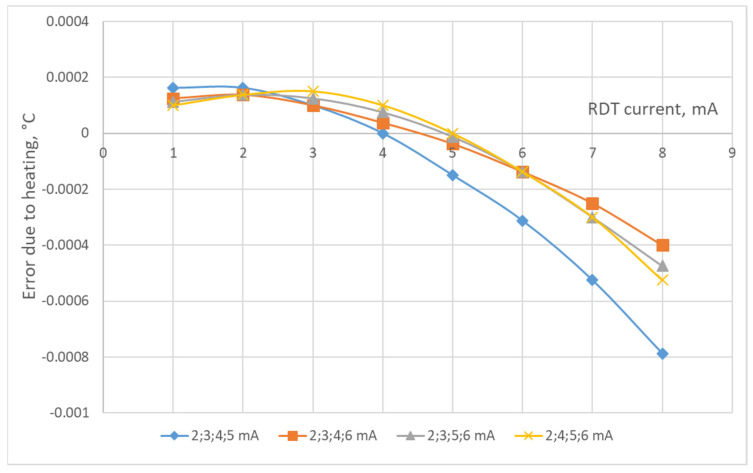
The maximum residual error for four measurements and the maximum ADC noise of 0.0004 °C for larger currents.

**Figure 6:**
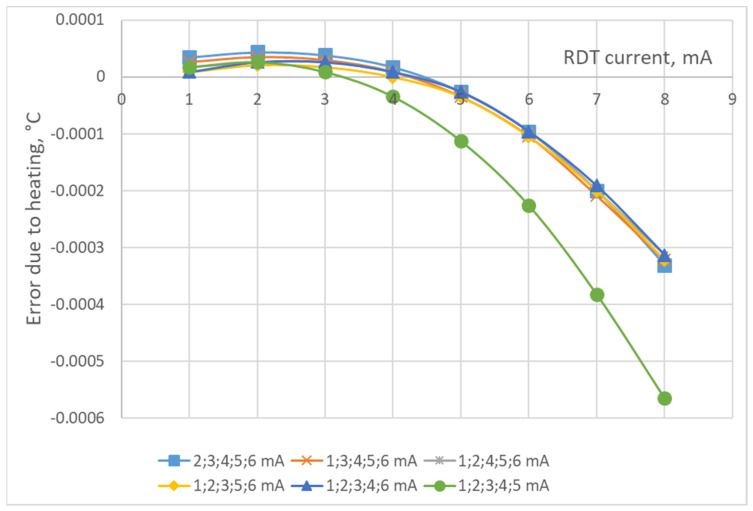
The maximum residual error for five measurements and the maximum ADC noise of 0.0002 °C.

The study of the residual error after correction of the error due to heating the RTD by the operating current was carried out by modeling. The research algorithm consists of calculations according to (9)–(15) for sets of the RTD’s currents from 1 to 10 mA under two levels of the maximum random ADC noise of 0.0002 °C (this slightly exceeds the previously determined minimum achievable noise of AD7714) and 0.0004 °C. To find the maximum residual error of the correction, its determination was carried out 100 times. The results of the study of the residual error after the correction of the error due to the heating of the P100-type RTD by the operating current, according to the proposed technique of determining the coefficients *A*_0_, *A*_1_, *A*_2_ for the correction polynomial for the corresponding currents, are presented in Figure 3, Figure 4, Figure 5 and Figure 6. As can be seen from these figures, all dependencies of the maximum residual error have a zone of small error, which corresponds to small operating currents of the RTD (the zone of measurements) and a zone of a significant rise in the error (the zone of the determination of coefficients *A*_0_, *A*_1_, *A*_2_). The measuring current should be set in the first zone, while its value, as can be seen from Figure 3, Figure 4, Figure 5 and Figure 6, can vary significantly. The currents for determining coefficients *A*_0_, *A*_1_, *A*_2_ should be chosen from the condition of minimum error and its changes during measurements. Figure 3, Figure 4, Figure 5 and Figure 6 also show that the correction error decreases with a decrease in the random error of the ADC and an increase in the number of currents at which the coefficients *A*_0_, *A*_1_, *A*_2_ (decreases almost by half) are determined.

It is possible to determine the coefficients *A*_0_, *A*_1_, *A*_2_ of the correction polynomial only under the following conditions:

1. The temperature of the measurement object remains stable throughout the process of determining the coefficients (the instability of temperature measurement readings does not exceed the noise of the ADC). Otherwise, there is a correction error associated with the superposition of the change in the object’s temperature and error due to heating the RTD. To monitor the stability of the object’s temperature, we can use either another RTD or a closed measurement cycle: the results of the measured temperatures are saved before the procedure of determining the coefficients of the correction polynomial, and these results are compared with the measurement results after the procedure of determining the coefficients of the correction polynomial. If these results differ by more than twice the maximum ADC noise, the results of determining the coefficients of the correction polynomial are not reliable and the whole procedure should be repeated.
2. The resistance of the RTD should be measured after the end of the thermal transience of heating by the operating current, otherwise, an additional error will occur. This error can considerably distort the results of determining the coefficients of the correction polynomial. The required waiting time can be determined from the analysis of the change in the measured temperature. It is necessary to set the waiting time equal to at least 20 time constants of the resistance thermometer.
3. The coefficients of the correction polynomial can be determined quite rarely. The fact is that in stationary operating conditions, the mode and parameters of heat exchange between the thermometer and the object of measurement change little. These changes will affect the residual error after the correction, that is, they will become the error from the error. Therefore, we can perform the above points 1 and 2 at the beginning of the operation of the temperature measurement system. Then we can use their results until significant changes in the operating mode of the object or the mutual placement of the RTD and the object happen. However, if these heat transfer changes are deterministic in nature, we can investigate the coefficients of the appropriate number of correction polynomials to be used under the appropriate conditions. The suitability of the proposed method for dynamic temperature measurements should be studied in each particular case.

## 4. Discussion

As demonstrated by the results of the conducted research, correcting the error caused by the heating of a platinum resistance thermometer (PRT) by its operating current can substantially reduce the influence of this error on the final temperature measurement results. Only under certain conditions is the reduction less effective. For instance, Figure 2 shows the error due to self-heating versus ADC noise and interference. We estimate the error due to self-heating to be 0.008 °C when using the ADC of AD7714. From Figure 3, Figure 4, Figure 5 and Figure 6, it can be seen that the residual error due to self-heating after correction does not exceed 0.0002 °C for measurement currents below 5 mA. The residual error after correction is about 0.001 °C for the worst case, i.e., when the measuring current is 8 mA and the error is estimated due to self-heating with currents of 2,3,4,5 mA (see bottom right corner of Figure 5). In all other cases, the reduction can be by a factor of ten or even more, which is a significant improvement in measurement precision. By applying certain methods, such as the least squares method, the residual, non-excluded error from PRT heating can be further minimized. This method allows for the error to be reduced to levels that are even smaller than the noise level of the analog-to-digital converter (ADC), which is an impressive outcome in precision measurement. Importantly, achieving this level of correction typically requires only five measurements. Increasing the number of measurements beyond this may only be necessary in specific, individual cases, depending on the particular measurement environment or accuracy requirements.

Achieving high accuracy in the correction of heating errors for the platinum RTD relies on the condition that the relative errors in resistance measurement are kept small under different operating conditions. When measuring temperature within a relatively narrow range—where changes in PRT resistance are relatively small—this condition can be met by using multi-bit sigma-delta ADCs. These types of ADCs are known for their ability to provide high-resolution readings and reduce measurement noise, which helps maintain small relative errors even in the presence of slight variations in resistance.

However, when temperature measurements need to be taken across a wide range—where PRT resistance changes more significantly—different strategies need to be employed to maintain accuracy. In such cases, it is advisable to use the substitution method. This method allows for a small relative error in resistance measurement at different PRT currents, which is particularly useful when determining the coefficients of the correction polynomial used to account for heating effects. The correction polynomial is essential for adjusting the temperature reading based on the influence of current-induced heating, ensuring that the measurement remains accurate.

Even if the measurement involves a reference resistor that is of relatively low accuracy, such as the C5-61 resistor model, the substitution method can still achieve highly accurate results. The key to this lies in the use of the substitution method in conjunction with a two-decade transient measure. This combination enables the measurement system to maintain a small relative error in PRT resistance readings, even at different current levels. The effectiveness of this approach is such that, in many cases, the small relative errors can be considered negligible, meaning they do not significantly impact the overall accuracy of the measurement.

Furthermore, this approach can achieve such high precision without the need to apply additional, complex correction methods that are often required to deal with the nonlinear error component of the ADC. Research has shown that the methods developed in prior studies, such as those outlined in [19], are not always necessary when the substitution method and two-decade transient measure are used effectively. This simplification can save time and effort in certain measurement scenarios, while still delivering high levels of accuracy.

## 5. Conclusions

The research confirms that the proposed method for correcting the error due to heating of the RTD can considerably improve measurement accuracy by reducing this error by an order of magnitude. The use of a multi-bit sigma-delta ADC is crucial. The least squares method, in particular, proves to be effective in reducing residual errors to a level well below the noise level of the ADC. Five measurements are enough for this. A further rise in the number of measurements can be reasonable only in special cases. The result is a highly accurate, reliable temperature measurement system that can function without the need for additional complex corrections. The proposed method can be useful for implementation in modern facilities of Industry 4.0.

## Data Availability

Data can be obtained on request from the corresponding author.
